# Activity, assay and target data curation and quality in the ChEMBL database

**DOI:** 10.1007/s10822-015-9860-5

**Published:** 2015-07-23

**Authors:** George Papadatos, Anna Gaulton, Anne Hersey, John P. Overington

**Affiliations:** European Molecular Biology Laboratory, European Bioinformatics Institute (EMBL-EBI), Wellcome Trust Genome Campus, Hinxton, Cambridgeshire CB10 1SD UK

**Keywords:** Public bioactivity databases, Data curation, Data quality

## Abstract

The emergence of a number of publicly available bioactivity databases, such as ChEMBL, PubChem BioAssay and BindingDB, has raised awareness about the topics of data curation, quality and integrity. Here we provide an overview and discussion of the current and future approaches to activity, assay and target data curation of the ChEMBL database. This curation process involves several manual and automated steps and aims to: (1) maximise data accessibility and comparability; (2) improve data integrity and flag outliers, ambiguities and potential errors; and (3) add further curated annotations and mappings thus increasing the usefulness and accuracy of the ChEMBL data for all users and modellers in particular. Issues related to activity, assay and target data curation and integrity along with their potential impact for users of the data are discussed, alongside robust selection and filter strategies in order to avoid or minimise these, depending on the desired application.

## Introduction

The ChEMBL database, provided as part of a broad range of life-science informatics resources at EMBL-EBI, is a key representative of a current plethora of publicly available chemical structure and bioactivity databases (which also include, for example, PubChem BioAssay, BindingDB, GuideToPharmacology and DrugBank) [1–7]. The emergence and increasing popularity of such databases has arguably democratised the fields of computational medicinal chemistry and chemical biology, and more generally drug discovery. Large-scale access to quality data for data-driven analyses of polypharmacology, bioisosteric replacements, chemogenomics, drug repurposing and predictive modelling are no longer the exclusive privilege of a few commercial organisations [8–14]. Moreover, ChEMBL’s open nature, as reflected in its clear data licensing terms, has brought about a dramatic shift in the way the drug discovery community deposits, shares and consumes experimental data, thus forging data exchange activities, collaborations and pre-competitive initiatives across industry, small and medium-sized enterprises (SMEs), charitable organisations and academia [15–18].

ChEMBL covers a broad range of curated and annotated data, mostly manually extracted from the primary medicinal chemistry literature. The data include experimental biological readouts, such as binding, functional, absorption, distribution, metabolism, and excretion (ADME), as well as toxicity assay measurements. Importantly, a curated linkage between indexed 2D chemical structures and biological targets is provided, whereby measurements are standardised to common types and units, where possible. The targets range from single proteins, to protein complexes, sub-cellular components, cell-lines, then tissues and finally whole organism in vivo data. In addition to the literature-extracted information, ChEMBL also integrates deposited screening results from PubChem Bioassay [4], along with information on approved drugs, late-stage clinical development candidate drugs, and their likely efficacy targets. ChEMBL also serves as an open data-sharing hub for the important field of neglected tropical diseases (NTD) research. The ChEMBL database is updated on a regular basis and, as of March 2015, the current version (version 20) contains more than 13 million experimentally derived bioactivities. For comprehensive reviews of ChEMBL with regard to content, coverage, availability and applications, the reader is referred elsewhere [1, 2, 19].

The increase in the use of ChEMBL, along with other bioactivity databases, was followed by an increase in the community’s awareness of the topics of data integrity and quality [20]. As a direct result, there have been several publications on such topics in the last few years. Even before the ChEMBL database was established, Fourches et al. [21] were among the first to systematically codify and document common sources of errors in public datasets and databases in the context of chemoinformatics and QSAR modelling research. Their review focussed on the quality of chemical structures and exemplified typical sources of errors and discrepancies such as treatment of tautomerism, aromaticity, salts and functional group standardisation. More recently, Tiikkainen et al. [22] have systematically analysed and compared the inconsistencies introduced during the extraction, digitalisation and subsequent curation of the data in three major commercial and publicly available sources of bioactivity data, including ChEMBL (version 14), Liceptor and WOMBAT. The authors looked at cases where, for the same bioactivity data point, two sources agreed in terms of ligand structure, biological target assignment, activity value and activity type but where the third did not. The analysis indicated that the most frequent source of discrepancies was the structure of the ligand, followed by the target assignment, the activity value and finally the activity type—this trend would be expected by the inherent complexity of the corresponding data objects. More importantly, all accurate discrepancies were communicated to the ChEMBL curation team and were corrected in subsequent releases of the database.

In addition to the errors introduced during the data extraction and digitalisation step, there are inherent discrepancies and ambiguities in the publications themselves. Some of these may be identified by means of automated large-scale data mining. Kramer et al. [23] analysed K_i_ data derived from ChEMBL (version 12) in order to quantify the experimental uncertainty of independent measurements for the same ligand–protein pair, and thus define the maximum unbiased performance of in silico models. During this effort, the authors identified several types of putative activity issues and errors, listed below:

- Unrealistically high or low activity values
- Multiple values for the same ligand–protein pair derived from a single publication
- Multiple citations of a specific activity value (exact or rounded) for the same ligand–protein pair across several publications leading to redundancy
- Unit transcription and conversion errors

Notably, some of these are not data errors *per se*: for example, the apparent existence of multiple measurements for the same ligand–protein pair within a paper often arises when authors have reported activity measurements for racemic mixtures, as well as for the isolated but structurally uncharacterised individual stereoisomers (e.g., Figure 3a in [23]). Similarly, repeat citations of single activity measurements across multiple publications might not be considered to be an error, but can nevertheless lead to statistical artefacts during the data modelling process. The issues listed above were communicated to the ChEMBL data integration team and were subsequently retrospectively flagged in the ChEMBL database, as described in detail in the sections below. In addition, the ChEMBL database schema and interface were appropriately updated to accommodate the results of this large-scale automated curation effort.

In a follow-up review, the same authors discussed data quality in bioactivity and chemogenomics databases [24]. In addition to the issues identified above, the authors highlighted cases related to inaccurate or insufficient target assignments, along with insufficient information in assay description, which prevents users from comparing measurements for the same ligand-target pairs across different assays. These topics are critically discussed in the following sections, whereby the focus in on the activity, assay and target curation and associated issues in the ChEMBL database. For a review and discussion on the compound curation and representation issues in public bioactivity databases, the reader is referred elsewhere [20, 28]. Table 1 summarises common types of errors and ambiguities found in bioactivity databases.

**Table 1:** Sources of errors and ambiguities related with bioactivity databases

Error source	Examples	References
Experimental	Compound purity and stability	[21, 25]
	Errors in compound vendor catalogues. Errors in cell-line identity	
Data extraction	Missing stereochemistry or functional group	[20, 26]
	Incorrect or incomplete target assignment	
Author of publication	Insufficient assay description. Citation of previously reported activity values	[23, 27]
	Wrong activity type and units. Incorrect data processing	
Database user	Merging activities from different assays	[21, 23, 28, 29]
	Dealing with censored data points, tautomers, prodrugs, salts and duplicates	

## Activity, assay and target curation in the ChEMBL database

**Fig. 1:**
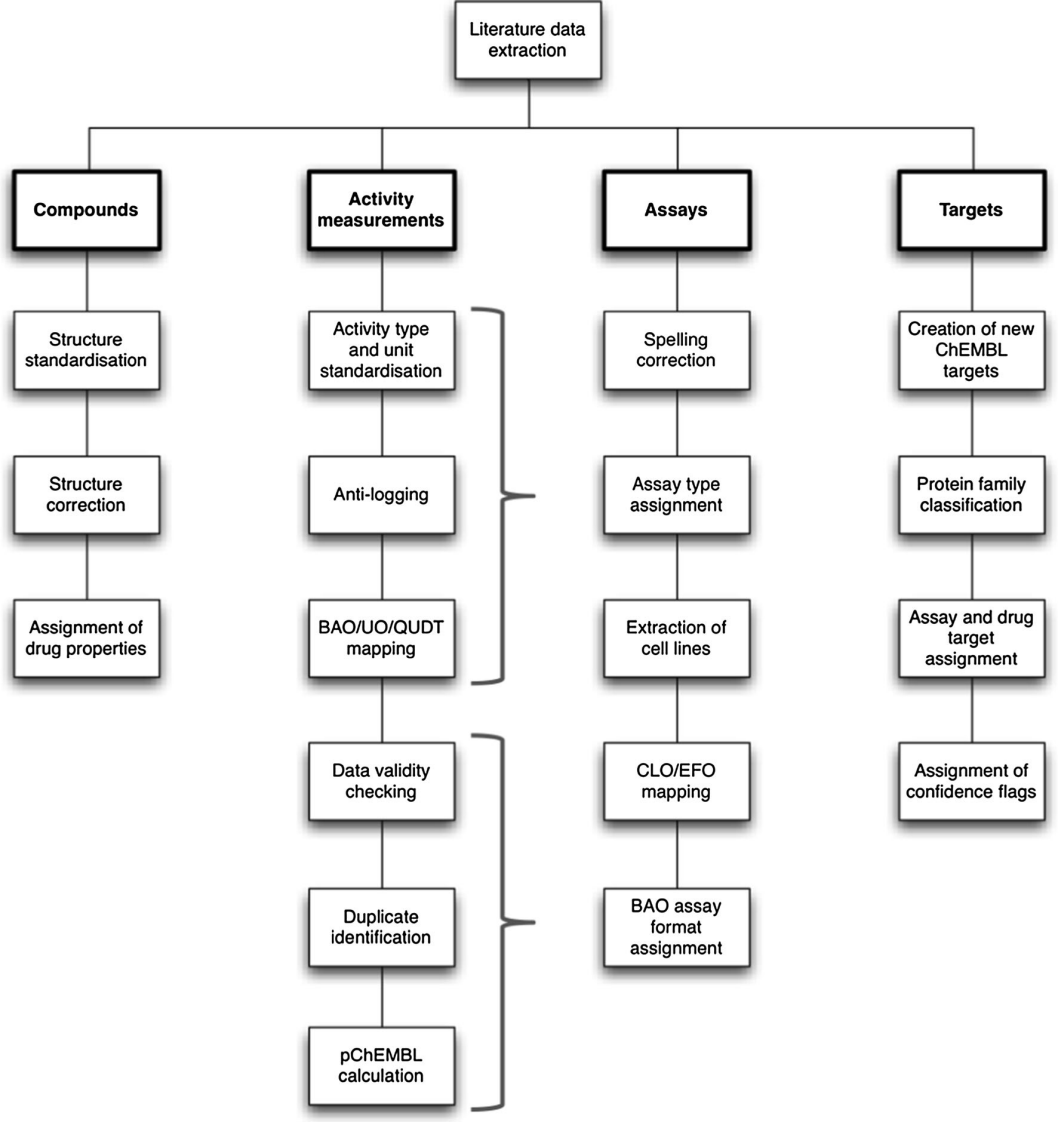
The current in-house compound, activity, assay and target curation workflow in ChEMBL production. The steps involved in the activity, assay and target curation branches, along with suggestions on how the users/modellers can utilise these to improve data integrity and minimise or avoid ambiguity are discussed in the following sections

The initial extraction of bioactivity data from the scientific literature is performed manually and aims to capture data as reported in a particular publication (i.e. the compounds, measurement types, units and values as provided by the author). The sections below provide an overview of the further manual and automated approaches that take place in-house and aim to standardise, curate, flag, map and annotate activity, assay and target data in ChEMBL. Furthermore, each of the respective sections is followed by a discussion on related data integrity and ambiguity issues and how these can be identified and minimised or avoided by the database user. A schematic summary of the steps currently involved in the curation of literature data in ChEMBL is provided in Fig. 1 below.

## The activity values curation process

**Fig. 2:**
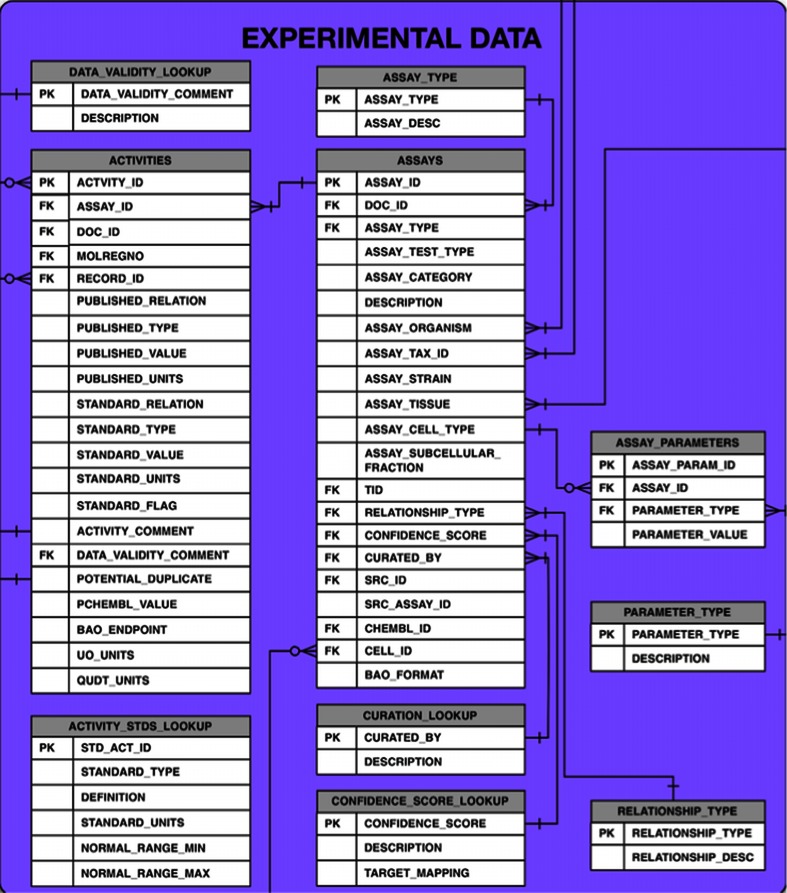
The experimental data section of the ChEMBL 20 database schema, showing the columns of the ACTIVITIES and ASSAYS tables

One problem faced in the extraction of published bioactivity data is the diversity of measurement and unit types used. For example, the ChEMBL measurement types IC50 and clearance have each been extracted from the literature associated with more than one hundred different published units. These activity types, units and values are respectively captured as ‘PUBLISHED_’ in the ACTIVITIES table (Fig. 2), in order to aid users in mapping data back to the original publication. However, comparison of data across different publications would then require the time-consuming and error-prone step of unit conversion by individual users. To address this need, a standardisation workflow has been designed and implemented. For key activity types in ChEMBL, a preferred unit or small set of units of measurement have been selected, as captured in the ACTIVITY_STDS_LOOKUP table (Fig. 2). Subsequently, extensive sets of unit conversion rules have been manually compiled and employed to standardise the data. IC50 and EC50 measurements, for instance, are converted either to nM or μg × mL^−1^ units, depending on the form of the original published units. Similarly, different descriptions of the same activity type (e.g., ‘Elimination half life’, ‘Half life’, ‘half-life’, ‘T1/2′, t1/2’, and ‘t(1/2)’) are also normalised to a single one. This activity type and unit standardisation step has enabled the conversion of activity values recorded in the literature with 133 different concentration units to consistent nM values. Likewise, AUC (Area Under the drug concentration time Curve) data with 83 units has all been standardised to units of ng × h × mL^−1^. Examples of the numbers of published to standard activity types and published to standard units are shown in Table 2a, b, respectively. Moreover, records with activity types such as pKi and logIC50 are converted to their unlogged standard value and corresponding unit and relation. Finally, the standardised activity types are mapped to BioAssay Ontology (BAO) result terms [30, 31] and the standardised units to unit ontology and quantities, units, dimensions and data types (QUDT) terms [32, 33]. These ontology mappings formalise the meanings of the activity types and units, preventing ambiguities in their interpretation, and also allow more advanced queries of the data, such as grouping different concentration–response endpoints based on BAO.

**Table 2:** Number of distinct published activity units (a) and activity types (b) mapped to standard, normalised units and types, respectively, after the standardisation step

Number of distinct published activity units	STANDARD_Unit
*(a)*	
133	nM
83	ng × h × mL^−1^
56	μg × mL^−1^
36	μM × h
28	mL × min^−1^ × kg^−1^
20	mL × min^−1^ × g^−1^
17	mg × kg^−1^
16	μmol × g^−1^
15	h
10	L × kg^−1^

Number of distinct published activity types	STANDARD_Type
*(b)*	
31	CL
29	AUC
21	T1/2
17	Vd
15	Solubility
14	IC50
10	Vdss
10	F
7	Kd
6	k_on

In addition to the standardisation and mapping effort described above, there has been a significant on-going effort to capture the activity curation knowledge and experience and distil it into a number of steps that can be run in an automated fashion. The aim of this workflow is twofold: (1) to flag activity records with potential quality issues, mainly introduced by the publication authors or extraction process; (2) to further standardise the activity records, thus making them more accessible and suitable for large-scale data mining and comparative analyses. The flags and comments are primarily added to the DATA_VALIDITY_COMMENT column in the ChEMBL database production schema (Fig. 2). Since ChEMBL version 15, the resulting workflow has been run before each ChEMBL release and further activity type and unit standardisations are regularly added to this auto-curation pipeline. The automated activity curation workflow is summarised in Table 3.

**Table 3:** The activity records curation workflow along with the count and percentage of affected records in ChEMBL 20

Order	Step	Data validity comment	Num. and % affected records
1	Flag missing activities	‘Potential missing data’	12,263—0.09 %
2	Flag non-standard units for activity type	‘Non standard unit for type’	81,060—0.6 %
3	Convert log activity values	N/A	2.6 × 10^6^—20.3 %
4	Flag out of range values	‘Outside typical range’	187,108—1.7 %
5	Flag potential duplicate values	N/A	64,860—0.48 %
6	Flag potential transcription errors	‘Potential transcription error’	382—0.003 %
7	Calculate standard negative log values	N/A	2.8 × 10^6^—20.7 %

The workflow starts with flagging missing data, i.e. data without a published activity value or activity comment. Then, records with unusual units for their respective activity types are flagged by looking-up an in-house curated list of standard types and their corresponding units. As an example, concentration-based activities, such as minimum inhibitory concentration (MIC) IC50 and K_d_, featuring irrelevant, unknown or null associated units are flagged in this step.

The third step involves the further standardisation of records with activity types, such as pKi and logIC50, which are converted to their unlogged standard value and corresponding unit, including exact and censored values. This affects more than 20 % of the activities in ChEMBL and ‘unlocks’ a significant number of data, which are then accessible and readily comparable for large-scale data mining. Furthermore, at this stage, the standard activity values that are lower than 10 are rounded arithmetically to 3 significant figures. The remaining values are rounded to the second decimal digit.

With the majority of the records having standardised values and units, the next step in the workflow flags records that have unusually low or high activity values for their type and unit. This is achieved by looking-up an internal list of normal value ranges, compiled and maintained by in-house curators. It should be noted that the defined normal ranges used in this process are fairly stringent (e.g., 0.01 nM–100 μM for IC50/Ki values) and some data falling outside of these ranges may be correct. A special case is made for binding affinities that involve *fragments* (MW < 350) as reported in relevant fragment screening publications; these are likely to be genuinely lower. Such cases are treated with more relaxed activity ranges.

The fifth and sixth steps aim to detect and flag duplicate entries and potential transcription errors in activity records that come from different publications, whereby a process similar to the one reported by Kramer et al. [23] is followed. Putative duplicated entries are records with identical compound, target, activity, type and unit values that were most likely reported as citations of measurements from previously published papers, even when these measurements were subsequently rounded. Transcription errors consist of otherwise identical entries, whose activity values differ by exactly 3 or 6 orders of magnitude, thus indicating a likely error in the units (e.g., μM instead of nM).

The final step of the workflow involves the calculation of the pChEMBL value (the negative logarithm of the activity values (in M units)) for records with dose–response activity types, such as IC50, XC50, EC50, AC50, K_i_, K_d_, and Potency and where there are no data validity comments.

## Activity records curation: discussion

Although the error rates in activity value and type are relatively lower than target mapping or structure representation [22], they may introduce ambiguity and be detrimental to the robustness of generated statistical learning models and activity summarisation efforts [23, 27]. Often for instance, the calculation of the median or mean activity value for a compound-target pair is the first step for the generation of a QSAR regression model. This summary figure will be significantly skewed by the presence of transcription errors and/or unrealistically low or high activity values. At the same time, confidence in an activity value may be overestimated due to citation-related over-sampling, as opposed to truly independent measurements.

It has to be highlighted here that errors such as duplicate values and unit transcription errors are sometimes inadvertently introduced by the authors of the publications. For example, it is common practice for authors in the medicinal chemistry literature to include tables in which they cite measurements for reference compounds, such as known tool compounds or drugs. These citations refer to previous papers often reporting different assay protocols and activity units, which are then misquoted in the table. This is clearly illustrated in the case of two publications [34, 35] from the same corresponding authors, who mis-cite a number of K_i_ activity values with the wrong units for two assays and several reference compounds (Table 1 in both publications). This has lead to otherwise matching activity records with 1000-fold activity value difference being recorded in the database. After the activity records curation step, the corresponding records from the later publication are flagged as ‘potential transcription errors’ in the DATA_VALIDITY_COMMENT of the ACTIVITIES table.

While manually checking all publications for cited duplicate values or transcription errors would be an impossible task, the existence of multiple measurements for the same compound and target facilitate automated approaches to identify such inconsistencies and outliers in the data. The automated activity curation workflow complements the manual curation performed by the in-house biological curation experts. Its aim is not to delete activity records but merely to flag potential and putative issues, inherently found in publications or potentially introduced by the data extraction process. By incorporating the flags in the DATA_VALIDITY_COMMENT and POTENTIAL_DUPLICATE columns in their analysis procedures, the interested user/modeller may adjust appropriately the granularity in the validity of the activity data, based on the scale and type of the desired data mining application; examples include local or global QSAR modelling (regression or classification), chemogenomics modelling or matched molecular pair analysis. As it has been suggested before [21, 26], data preparation and filtering is equally, if not more important, than the analysis or model itself.

In parallel, additional annotation with the pChEMBL (negative logarithm of activity in M) value adds more coverage and leverage to the activity data; this allows for comparisons across assays, publications and activity types for a given compound-target pair, which is suitable for larger scale data integration and modelling, such as ligand-based target prediction [9, 36, 37]. In the case of mixing heterogeneous pIC50 and pK_i_ data, there is evidence that this does not lead to loss of quality, given that certain criteria are met [27].

## The assay curation process

Curation of assays in ChEMBL focuses on capturing key characteristics of the experiment that has been performed (such as the assay type, format and cell-line/tissues used) and the assignment of targets (discussed in the next section). Spelling correction is first performed on assay descriptions in order to correct commonly misspelled words and therefore improve search and query recall. Next, each assay is assigned an ASSAY_TYPE which can take one of five values: B (binding), F (functional), A (ADME), T (toxicity) and P (physicochemical). Since these categories are not necessarily mutually exclusive, an order of precedence may be applied. For example, a cytochrome P450 3A4 binding assay could potentially be assigned a type of either B or A, but is generally assigned to the ADME category, as this is considered to be more informative in allowing users to identify and filter relevant data. Similarly, a cytotoxicity assay could be either a functional assay (in the context of a cancer) or a toxicity assay (when assessing whether a compound has adverse toxic effects). Cell-lines used in assays are extracted from assay descriptions and mapped (in the ChEMBL CELL_DICTIONARY) to existing published ontologies, such as the experimental factor ontology (EFO) and the cell line ontology (CLO) [38, 39]. This process can be complicated by the fact that cell lines may not always be adequately described in the original publication in order to allow unambiguous identification. The term ‘H4’, for example, can variously be used to refer to either a rat hepatoma cell line (ATCC CRL-1548), or a human neuroglioma cell-line (ATCC HTB-148). Work is also under way to extract organ/tissue information for assays and map to the appropriate Uberon ontology terms [40]. Finally, a rule-based classifier is used to determine the assay format, according to the BioAssay Ontology (e.g., biochemical, cell-based, tissue-based, organism-based).

## Capture of assay details: discussion

A frequent criticism of the ChEMBL database is a lack of detail captured regarding the assay protocols. Such information is important as it allows users to reliably compare assays conducted in different labs for the same compound or biological target. While key details of the assay are typically captured in the assay description, the need for more structured representation of such data is recognised. Therefore, a mechanism to allow more robust capture of these in the ASSAY_PARAMETERS database table (Fig. 2) has been recently implemented, in anticipation of future, more complete bioassay reporting [41]. Information such as the concentration or dose at which a compound has been tested, administration route for an in vivo assay, or time point at which the measurement was taken can now be captured, where such information is available. Details such as mutations within the protein target (which could greatly affect compound activity) or the use of particular assay constructs such as chimeric proteins could also be captured in this way. Currently, the target assigned in ChEMBL represents the full-length, wild-type protein but details of mutations may be captured in the assay description. An example of one of the limitations here is that of thrombin for which the initially produced protein is in a pre-pro form, i.e. has a secretion signal, and after cleavage of this signal sequence, circulates in a catalytically inactive form, in which ligand binding for most classes of inhibitors does not occur. Additionally, activity of mature thrombin, further depends on various ion-binding and post-translational amino-acid modifications. Future plans also include the extraction of assay parameters from existing assay descriptions by means of text mining, where possible, so that they can be queried more effectively, as well as seeking to capture these details for future additions to the database.

All the annotations described above will all aid users in filtering the ChEMBL data to the kind of assays they are interested in (e.g., retrieve all organism-based ADME assays with a particular activity type such as clearance, or all cell-based cytotoxicity assays using mouse 3T3-L1 cells). However, it should be noted that it will still likely not be possible to annotate assays to the level of detail that would allow determination of whether two independently conducted assays are truly identical in protocol (for example the detection instrument with which the measurements were taken, the full composition of the buffer used). Many of the journals from which data are extracted do not require details of assay protocols to be included where these have previously been published. Therefore, for a given article, the relevant information may be contained in another cited article (which may, in turn, cite other earlier publications). While essential assay details are extracted from these cited publications, where available, to extract more fine-grained assay details at large scale would be prohibitively time-consuming and expensive. It should also be noted that even when assay conditions appear to be the same, significant variability is observed between measurements taken in different labs [27].

For the reasons described above, users should therefore consider the assay annotation provided in ChEMBL as a filter to remove assays that are clearly not comparable, rather than a guarantee that two assays are comparable. It is always advisable to consult the original publications and obtain further information regarding the experimental setup where such details are considered of great importance. On the other hand, for large-scale applications, the size of the data set may be sufficient that division of assays into broadly similar subsets (e.g., biochemical vs. cell-based) may be robust enough to yield useful results.

## The target curation process

**Fig. 3:**
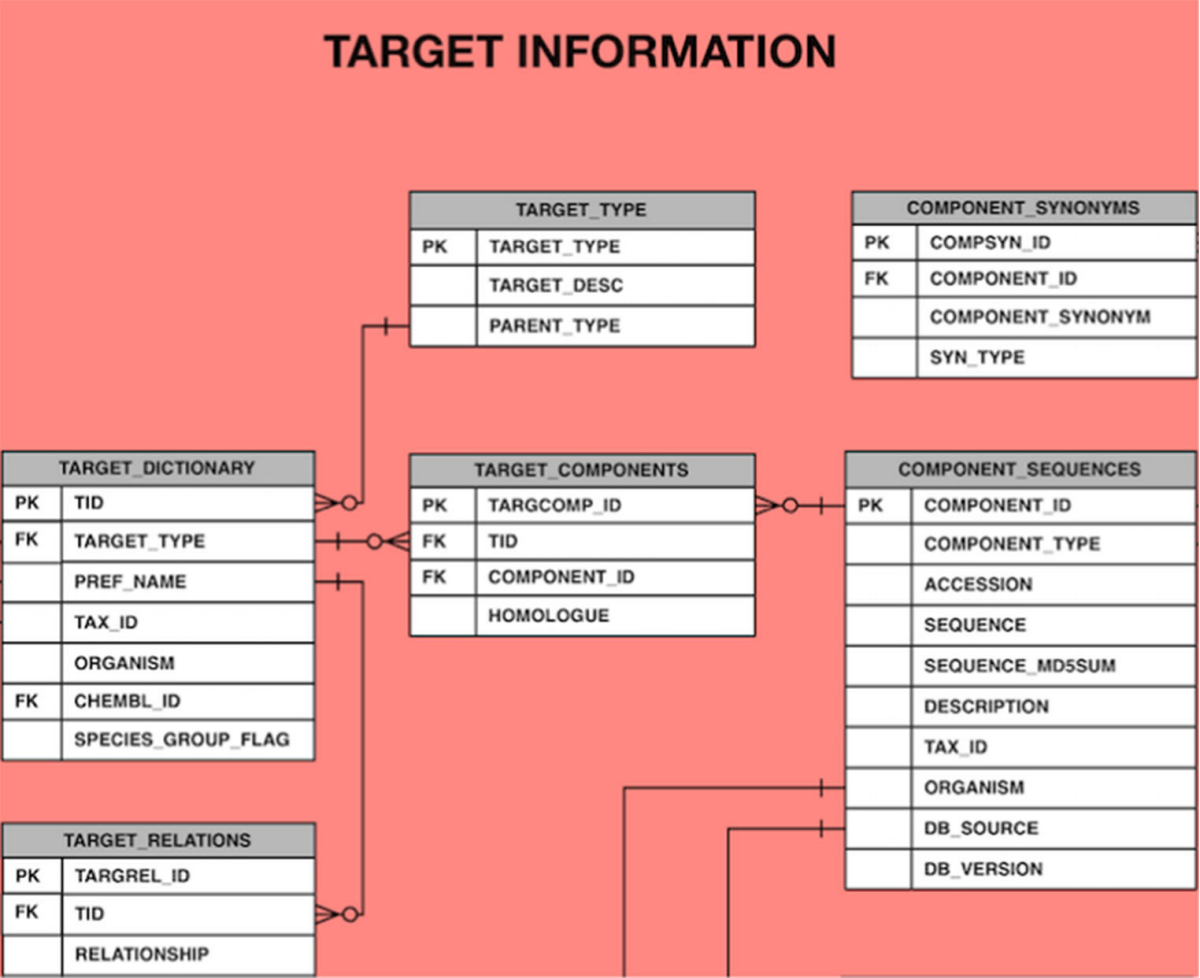
A subset of the target information section of the ChEMBL 20 database schema

To achieve one of the main aims of ChEMBL, i.e. coupling ligand-regulated phenotypic effects to the genotype, it is necessary to assign each assay to a target, wherever possible. Within a publication, the target of an assay is usually only referred to by a name or abbreviation, rather than a database identifier. Additional curation effort is therefore required to determine the molecular identity of the target and assign this a unique ChEMBL target identifier listed in the TARGET_DICTIONARY table (Fig. 3). While UniProt is used as the reference source for protein sequences and identifiers within ChEMBL [42], an important distinction between the concept of a ‘protein’ and a ‘target’ is made: a target is defined as the entity with which the compound actually interacts in a particular assay system, which could be a protein complex, or a non-protein target such as DNA, for instance. This requires the creation of unique identifiers for these targets. While identification of the correct target is relatively trivial for some kinds of assays, in other cases the assignment can be much more complex. Within ChEMBL, a range of different target types are captured, depending on nature of the molecular entity assigned and confidence in assigning it. The criteria for assigning some of the common target types are discussed below. Each target is then associated with each of its individual molecular components (usually proteins) and these components are further annotated with information such as the protein family to which they belong, in order to facilitate searching and further grouping of the data.

## Biological complexity in target assignment: discussion

For a binding assay carried out on a single, isolated protein in vitro, it is usually trivial to unambiguously assign the correct target. However, such data represent only around 10 % of the activity measurements in the ChEMBL database. Far more common are functional assays carried out in cell-lines, tissues or whole organisms. In these complex systems the activity of a tested compound may be much harder to interpret, yet will also yield more valuable information regarding the efficacy of the approach. In cell-based assays, a target of interest is often overexpressed or the assay may be carried out using a competing ligand with known selectivity for that target. In these cases, the intended molecular target of the assay may be assigned, although this assignment should still be interpreted with some caution, as there could be other proteins or pathways within the cell-line contributing to the observed effect. In many other cases though, the molecular target responsible for the effects of the compound cannot be unambiguously determined. For example, a common assay for muscarinic acetylcholine receptor-mediated smooth muscle contraction involves the use of guinea pig ileum. While the M3 receptor subtype is now believed to be primarily responsible for this effect [43], this information was not known at the time many of these assays were performed, and other receptor subtypes are also present in the tissue—notably M2 receptors, which are more abundant than M3 receptors [44]. Similarly, many other functional assays potentially measure activity against a group of closely related proteins, thus identification of the receptor subtype(s) or enzyme isoform(s) contributing to the effect may not be possible. Mapping such assays to a single protein may be an oversimplification of the biology involved, while mapping the assay to each of the individual protein family members (as was originally done in ChEMBL) may mislead users, giving the impression that each subtype has been tested individually (though even in earlier versions of the database, a flag was present to allow users to detect this ‘multiple’ mapping). To deal with such situations where one cannot be sure of the precise molecular identity of the target, ChEMBL assigns a ‘PROTEIN FAMILY’ target, incorporating each of the possible family members. Annotating assays in this way still allows retrieval of potentially valuable data for users interested in a particular protein or protein family and wishing to include functional/phenotypic endpoints in addition to binding measurements, but also allows the exclusion of such data where a precise mapping is important (for example, training predictive models or assessing compound selectivity).

An additional level of complexity originates from the fact that many of the molecular targets of bioactive compounds are not single proteins but protein complexes. While in some cases it may be possible to identify the subunit of the complex to which the compound binds, in other cases compounds may bind to the interface between two subunits, or the identity of the binding site may not be known. Even when the binding subunit is known, for many applications it may be important to understand the composition of the full protein complex. For example, activity may only be observed in a functional assay if all of the required subunits are present, potentially leading to false positive results if this information is omitted. Again, mapping an assay where the target is a protein complex to each of the individual subunits could be misleading to users, suggesting that the compound might have activity against each of the subunits in isolation. This could confound various analyses, such as the assessment of druggability. Therefore such assays are mapped to a ‘PROTEIN COMPLEX’ target in ChEMBL. Furthermore, the data model also allows for the annotation of the binding subunit (or also a structural domain within a subunit) within the target, where known; this is annotated for targets of approved drugs.

It is also important to note that the two situations described above are not mutually exclusive and it is quite possible (and indeed common) to have activity measured in a cell or tissue-based assay where the intended target is known to be a protein complex, but the precise subunit composition is unknown. Perhaps the most common example of this is the measurement of binding to GABA-A receptors in rat brain membranes. GABA-A receptors are pentameric complexes consisting of various combinations of alpha, beta and gamma subunits. Furthermore, there are six subtypes of alpha subunit, three subtypes of beta subunit and three subtypes of gamma subunit, making a large number of different receptors possible. Both alpha and beta subunits are necessary for binding of the endogenous ligand, GABA, while many GABA-A receptor drugs (benzodiazepines) bind at alpha/gamma subunit interfaces. Certain receptor combinations appear to be restricted to discrete areas of the brain, while other regions express a diversity of receptor types [45]. Such cases are assigned a target type of ‘PROTEIN COMPLEX GROUP’ in ChEMBL, indicating that the caveats associated with both the ‘PROTEIN COMPLEX’ and the ‘PROTEIN FAMILY’ target type apply.

A further challenge in target assignment can be the identification of suitable protein sequences for inclusion. Not all species that have bioactivity data in ChEMBL have been fully genome-sequenced, and therefore it might not be possible to identify the correct protein in UniProt. In such cases an orthologous protein might be substituted (e.g., a human sequence in place of another mammalian target, or a model organism, such as *E. coli*, in place of another bacterial species). A RELATIONSHIP_TYPE field in the ASSAYS table (Fig. 2) indicates cases where the target assigned is a homologue (‘H’) of the actual assayed target. Again, it may be appropriate for users to filter out such data in certain circumstances.

In analysing and using ChEMBL data, it is therefore important for the user/modeller to consider which types of data are suitable for addressing the question of interest, to select only appropriate target types and to understand the relationship of these targets with each of their individual protein components. Users should also be aware that other databases integrating data from ChEMBL, whose data models differ, might not represent or display this, or other data in the same way.

## Conclusion and future outlook

The availability of public chemistry and bioactivity databases, along with large scale data-driven applications has increased the community’s attention to data curation and integrity issues, such as structure quality, name-to-structure fidelity, structure–activity mapping, activity data accuracy, assay description sufficiency, target assignment, author errors and redundancy. Better quality data mean more and higher confidence assertions and therefore more robust applications and models. In this work, the focus was on the activity, assay and target curation and associated issues. Current strategies to map, standardise, flag and further annotate the data were presented, along with recommendations on good practice when mining these. Notably, the efforts reported here are only a subset of the on-going in-house manual and automated curation, which include compound structures, compound synonyms and drug information. Future plans include more detailed extraction of terms and parameters from the assay description, standardisation of additional activity types (particularly in the areas of pharmacokinetic, toxicity and crop protection data) and the addition of further BAO mappings, such as the bioassay class (e.g., radioligand-binding assay, cell growth assay, cytotoxicity assay), facilitating more granular queries particularly against phenotypic assays. From a technical point of view, we aim to streamline the curation process, in order to accelerate the ChEMBL release cycles and enable better data validation and easier data depositions by users. Finally, in the longer run, we envisage broadening the access and scope of the curatorial process by providing a simple data-quality feedback mechanism in the ChEMBL interface, and eventually developing a publicly available curation interface. Such an interface could serve as a platform to curate either ChEMBL data, perhaps even in a crowd-sourcing manner, or in-house, proprietary data, according to standardised curation rules. Finally, some of the sources of potential error the and accompanying burden of curation could be reduced in future by the enforcement of standards for data recording [41] and mandates by journals to deposit bioactivity data in a public repository at the point of publication.
